# Development of castration resistance in prostate cancer patients treated with luteinizing hormone-releasing hormone analogues (LHRHa): results of the ANARESISTANCE study

**DOI:** 10.1007/s00345-022-04108-x

**Published:** 2022-09-04

**Authors:** J. C. Angulo, J. P. Ciria Santos, A. Gómez-Caamaño, R. Poza de Celis, J. L. González Sala, J. M. García Garzón, J. A. Galán-Llopis, M. Pérez Sampietro, V. Perrot, J. Planas Morin, on behalf of ANARESISTANCE study group, José Manuel Abascal, Víctor Barrond, Antonio Benedicto, Ana Carballo, José Ramón Cortiñas, Manuel Fernández, Eduardo Ferrer, Pablo Luís Guzmán, Miguel Ángel López, José Carlos Martínez, Carlos Olivier, Paula Peleteiro, Pedro Julio Pérez, Daniel Pesqueira, José Ponce, Manuel Ruibal, Josep Segarra, Eduardo Solsona, José Francisco Suárez, José Rosa, Ángel Tabernero, Félix Vesga, Almudena Zapatero

**Affiliations:** 1 Clinical Department, Universidad Europea de Madrid; Hospital Universitario de Getafe, Madrid, Spain; 2 Radiation Oncology Service, Hospital Universitario Donostia, Donostia, Spain; 3 Department of Radiation Oncology, Hospital Clínico Universitario de Santiago de Compostela, Santiago de Compostela, Spain; 4 Department of Radiation Oncology, Hospital Universitario Araba, Gasteiz, Spain; 5 Urology Service, Corporació Sanitària Parc Taulí, Sabadell, Spain; 6 Urology Service, Hospital General de Llerena, Llerena, Spain; 7 Urology Service, Hospital General Universitario de Alicante, Alicante, Spain; 8 Ipsen Pharma, S.A.U, Barcelona, Spain; 9 Urology Service, Hospital Universitario Vall d’Hebrón, Barcelona, Spain

**Keywords:** Gonadotropin-releasing hormone, Castration-resistant prostatic neoplasms, Prospective study, Observational study

## Abstract

**Purpose:**

Evaluate the percentage of patients with prostate cancer treated with luteinizing hormone-releasing hormone analogues (LHRHa) that develop castration resistance after a follow-up period of 3 years. The secondary objective is to evaluate the variables potentially related to the progression to castration resistant prostate cancer (CRPC).

**Methods:**

A post-authorization, nation-wide, multicenter, prospective, observational, and longitudinal study that included 416 patients treated with LHRHa between 2012 and 2017 is presented. Patients were followed for 3 years or until development of CRPC, thus completing a per-protocol population of 350 patients. A Cox regression analysis was carried out to evaluate factors involved in progression to CRPC.

**Results:**

After 3 years of treatment with LHRHa 18.2% of patients developed CRPC. In contrast, in the subgroup analysis, 39.6% of the metastatic patients developed CRPC, compared with 8.8% of the non-metastatic patients. The patients with the highest risk of developing CRPC were those with a nadir prostate-specific antigen (PSA) > 2 ng/ml (HR 21.6; 95% CI 11.7–39.8; *p* < 0.001) and those receiving concomitant medication, most commonly bicalutamide (HR 1.8; 95% CI 1–3.1, *p* = 0.0431).

**Conclusions:**

The proportion of metastatic patients developing CRPC after 3 years of treatment with LHRHa is consistent with what has been previously described in the literature. In addition, this study provides new findings on CRPC in non-metastatic patients. Concomitant medication and nadir PSA are statistically significant predictive factors for the time to diagnosis of CRPC, the nadir PSA being the strongest predictor.

**Supplementary Information:**

The online version contains supplementary material available at 10.1007/s00345-022-04108-x.

## Introduction

Prostate cancer (PC) is the second most common cancer, and the fifth most common cause of cancer-related mortality among male patients, worldwide. In Europe, the incidence of PC in men in 2020 exceeded that of lung cancer [1]. In developed countries, incidence is higher, possibly due to greater availability of prostate-specific antigen (PSA) testing [2, 3], and despite a significant mortality decrease (52% since 1993), PC remains the second cause of cancer-related death [2].

Androgen deprivation therapy (ADT) is the standard care for locally advanced and metastatic disease [4, 5]. PC cells require testosterone (T) and undergo apoptosis when lacking androgenic stimulation. T levels can be lowered with bilateral orchiectomy or administering LHRHa and antiandrogens. As bilateral orchiectomy has a potential psychological impact, drug therapy benefits outweigh the cost savings of surgery [5]. Complete androgen blockade (CAB) adds a non-steroidal antiandrogen (bicalutamide, flutamide, nilutamide) to therapy to inhibit adrenal androgens [6].

Although most PC patients respond to ADT, tumor cells may become androgen independent in 2–3 years [7, 8]. Multiple molecular mechanisms contribute to castration-resistant PC (CRPC), mostly involving the androgen receptor (AR) signaling pathway as an adaptative response to ADT [9]. In CRPC, PSA levels continue to rise in a low T level environment. Mortality of CRPC patients is high [10]. Despite several systemic therapies have demonstrated a survival advantage in metastatic castration resistant prostate cancer median overall survival based on real-world data may not exceed 2 years [10, 11]. Evidence shows that T levels < 20 ng/dL improve CRPC patient survival and delay disease progression [5, 9, 12]. Regular PSA and T level determinations predict treatment response and duration as well as disease progression, allowing for new strategies to be introduced [13–15].

Classical data revealed that only 7% of metastatic cancer patients treated with hormonal therapy were alive after 10 years with a mean response duration of 2.5 years [16]. Despite accumulating experience and knowledge of CRPC it is not well known how CRPC or median time to diagnosis of CRPC are influenced by epidemiological factors. The aim of this study is to evaluate the percentage of PC subjects in treatment with LHRH analogues who show castration-resistance state after a follow-up period of 3 years and to identify factors affecting poor prognosis.

## Methods

### Study design

ANARESISTANCE (Study number A-92-52014-204, Vall d’Hebron Hospital CEIm) was a post-authorization, prospective, observational, longitudinal study performed at 28 sites in Spain between 2012 and 2017. Prospective data were collected at four visits over 3 years: a baseline visit plus three visits 12 month apart. T and PSA levels were measured at each visit at local laboratories. We evaluated the percentage of LHRHa-treated PC patients developing CRPC, and identified factors related with CRPC development.

### Patients

Eligibility criteria were age 18 years or older, histological confirmation of PC and suitability to ADT with any LHRHa according to the summary of product characteristics (SmPC) for at least 2 years or having initiated LHRHa within 1 year of inclusion. Exclusion criteria were participation in another clinical study, less than 3 years life expectancy and intention of intermittent ADT at inclusion or during the first 6 months. LHRHa selection and use followed routine clinical practice.

The study population included all patients who provided written informed consent and started LHRHa treatment. The per-protocol population included LHRHa-treated patients followed for 3 years after baseline with no major protocol deviations. Stratification based on metastatic status was performed at baseline. The indication for treatment with LHRHa in non-metastatic cases was adjuvant treatment to radiation therapy in high-risk patients and old symptomatic patients not-considered candidates or reluctant to local treatment.

### Outcomes

The primary endpoint was the proportion of patients developing CRPC after 3 years of LHRHa. Secondary endpoints were median time to CRPC diagnosis and factors related to CRPC development. CRPC was defined as a serum T level < 50 ng/dl or < 1.7 nmol/L plus three consecutive increases of PSA, 1 week apart, resulting in two 50% increases over the nadir and PSA level > 2 ng/dl and anti-androgen withdrawal for at least 4 weeks (in the case of flutamide) and 6 weeks (in the case of bicalutamide) when used [17]. Furthermore, we aimed to estimate the median LHRHa treatment time until castration resistance. We evaluated whether age, race, PC personal and family history, stage (TNM classification), Gleason score, d’Amico risk classification, relapse, serum PSA level, serum T level and ECOG status influenced the time to castration-resistance.

The primary analysis was performed on the per-protocol population. Secondary analyses were conducted on the full study population.

### Statistical analyses

We used SAS^®^ software, version 9.4. The primary analysis was presented as absolute number and percentage of patients with an exact 95% confidence interval (CI). Time to CRPC was represented on Kaplan–Meier plots.

In the secondary analysis, a Cox regression model was used to identify factors potentially related to CRPC at 5% level. Predictive factors were identified by univariable and multivariable Cox regression analyses using a stepwise model with *p* = 0.2 entry and *p* = 0.05 stay criteria. PSA and T levels were analyzed by visit on the study population. Median T levels at visits 2, 3 and 4 were compared with median baseline T levels and among visits (visit 4 vs. visits 2 and 3) using the Wilcoxon Signed Rank test.

## Results

**Fig. 1:**
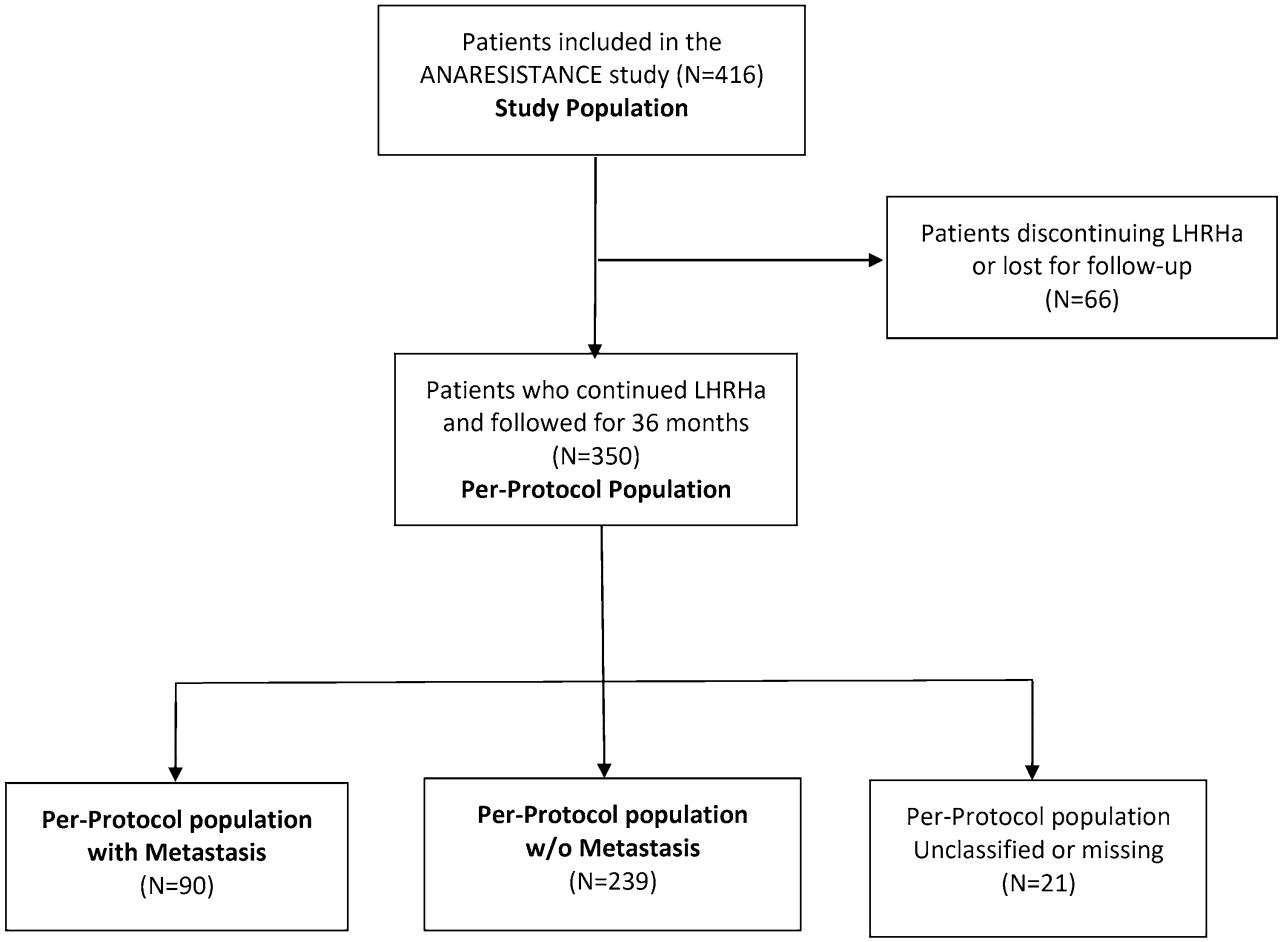
Flowchart of the population in the study

Patient distribution is presented in Fig. 1. Patients who signed the informed consent and received at least one dose of an LHRHa composed the study population (*n* = 416). Forty-one patients were lost to follow-up (9.9%), and eight patients withdrew consent (1.9%). CRPC status was non assessable in 66 (15.9%) patients who were not included in the per-protocol population, defined as patients who received LHRHa for 36 months in whom development of CRPC was assessed (*n* = 350).

### Characteristics of the study population

Mean ± SD age of the 416 patients in the study population, was 72.8 ± 8.18 years. The mean time between initial PC diagnosis and enrollment in the study was 15.6 ± 30.95 months. In 177 patients (42.5%) time since PC diagnosis to study enrollment was under 3 months. PC was diagnosed by screening in 274 patients (66%), the most frequent diagnosis method was PSA lab assay, (246; 89.8%). 38 (9.1%) patients had a family history of PC. Most patients had a good functional status (~ 60%, ECOG score = 0, ~ 25%, ECOG score = 1, at all visits). The most frequent comorbidities were cardiovascular in 191 patients (45.9%), metabolic in 118 (28.4%), renal in 65 (15.6%) and gastrointestinal in 43 (10.3%). During the study, 28 deaths were reported, none appeared related to the study drug.

Prostatectomy was performed in 61 patients (14.7%) and 83 (20%) received radiotherapy by baseline. In 199 patients (47.8%), LHRHa had been initiated before enrollment, and in 122 patients (30%) LHRHa was part of CAB. 379 patients (91.1%) received triptorelin 22.5 mg. Other LHRHa used were leuprolide in 32 (7.7%) and goserelin in 5 (1.2%). A total of 67 subjects (16.1%) received concomitant medication; the most commonly used was the antiandrogen bicalutamide (in 60 of 67 patients). Other concomitant medications included therapeutic radiopharmaceuticals (ten cases), abiraterone (three cases) and enzalutamide (three cases) and docetaxel (1 case). LHRHa was discontinued in 84 patients (20.2%) at a mean ± SD of 25.5 ± 10.6 months, never due to tolerability issues. LHRHa was discontinued due to exitus in 5 patients (6%), disease progression in 2 (2.4%), patient decision in 7 (8.3%), intermittent androgen blockade in 12 (14.3%), performance of radical prostatectomy) in 2 (2.4%), end of adjuvant radiation protocol in 52 (61.9%) and unknown cause in 4 (4.8%).

The baseline distribution of the study population according to TNM stage [18], Gleason score and T level is detailed in Table 1. Patients initiated on LHRHa at or after inclusion had higher T levels than those initiated before inclusion. Mean ± SD PSA level was 17.9 ± 36.69 ng/ml at screening visit (*n* = 400, 96.2%), and 23.2 ± 40.88 ng/ml before initiation of LHRHa (n = 286, 68.75%). Mean ± SD nadir PSA was 1.0 ± 5.71 ng/ml (*n* = 372, 89.4%): ≤ 2 ng/ml in 351 patients (94.4%) and > 2 ng/ml in 21 (5.6%). Mean ± SD time to the nadir PSA was 15.2 ± 9.64 months.

**Table 1:** Baseline patient distribution in the study population according to TNM stage, Gleason score, PSA and testosterone levels

	Study population (*N* = 416) (%)
Stage of disease (TNM staging system),* n* = 407	
Localized (T1-T2), N0, M0	118 (29.0)
Locally advanced (T3-T4-Tx or N1, M0)	154 (37.8)
Metastatic (M1)	114 (28.0)
Unclassified	21 (5.2)
Total Gleason Score,* n* = 413	
≤ 6	75 (18.2)
7 (3 + 4)	82 (19.9)
7 (4 + 3)	86 (20.8)
≥ 8	170 (41.2)
PSA levels (ng/dL),* n* = 400	
≤ 20	314 (78.5)
> 20	86 (21.5)
Testosterone levels (ng/dL),* n* = 284	
< 20	84 (29.6)
20– < 50	45 (15.8)
≥ 50	155 (54.6)
Testosterone levels (ng/dL), LHRHa initiated before inclusion,* n* = 141	
< 20	53 (37.6)
20- < 50	34 (24.1)
≥ 50	54 (38.3)
Testosterone levels (ng/dL), LHRHa initiated at/after inclusion,* n* = 143	
< 20	31 (21.7)
20- < 50	11 (7.7)
≥ 50	101 (70.6)

### Evolution of PSA and testosterone levels

In the study population, mean PSA levels decreased for 24 months and remained stable at month 36 (Appendix B, Supplementary Material). Mean T level decreased at month 12 but increased at months 24 and 36. LHRHa-treated patients not reaching castration levels were 13.2%, 15% when castration level was set at 20 ng/dl.

Median ± interquartile range (IQR) T levels were lower at visits 2 (*p* < 0.0001), 3 (*p* < 0.0001) and 4 (*p* < 0.0001) than at screening, ranging from 120.5 ± 387 ng/dl at screening to 20.3 ± 57 ng/dl at visit 4. Notably, median ± IQR values were higher at visit 4 than at visits 2 and 3 (20.3 ± 57 ng/dl vs. 15.0 ± 26 (*p* < 0.0001) and 17 ± 19 ng/dl (*p* < 0.0001), respectively) (Appendix C, Supplementary Material).

### Development of castration-resistance status

Since 64 (18.3%; 95% CI 14.38–22.74%) out of the 350 patients in the per-protocol population developed CRPC in 3 years, the median time to CRPC was non assessable. CRPC occurred in 36 of 90 metastatic patients (40%, 95% CI 29.81–50.87%) vs. 21 of 239 non-metastatic patients (8.8%, 95% CI 5.52–13.12%) (See in the Appendix A of the Supplementary Material a detailed distribution of CRPC status in the per protocol population at different times during follow-up and according to metastatic/non-metastatic and CAB status). Patients receiving CAB showed a trend to a higher rate of CRPC compared to patients not under CAB (21.6 vs 17.5%, 95% CI 14.04–30.81 and 12.91–22.91%). Of the 58 relapsing patients, 14 (24.1%, 95% CI 13.87–37.17%) developed CRPC, compared to 28 (18.9%, 95% CI 12.95–26.17%) of the 148 non-relapsing patients (relapse information was unavailable for 145 patients).

**Fig. 2:**
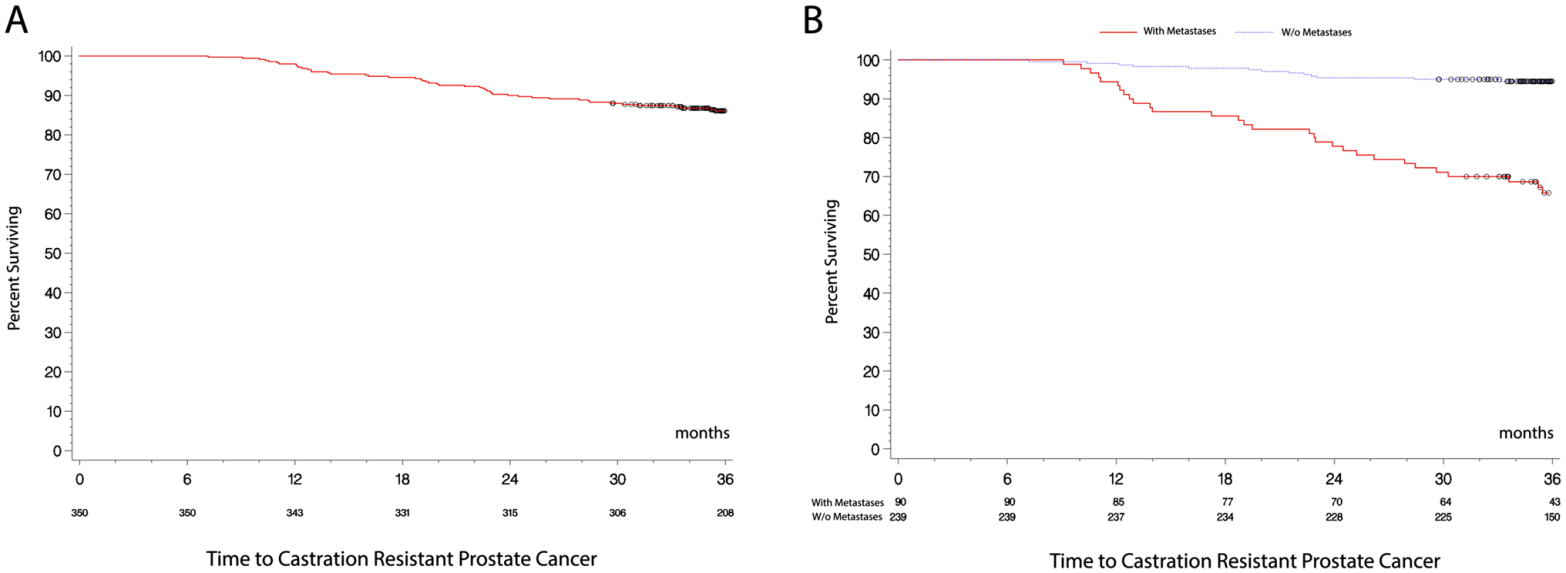
Kaplan–Meier curves for time from LHRHa treatment date to Castration-Resistant Prostate Cancer (CRPC) diagnosis, per-protocol population (**A**) and stratification for metastatic vs non-metastatic patients (**B**)

Although the median time to CRPC was non assessable, the 25th percentile of the time to CRPC diagnosis for the metastatic population was 28.5 (22.6–36.9) months (25th percentile non evaluable for non-metastatic patients). Figure 2 shows Kaplan–Meier curves for the time between LHRHa initiation and CRPC development.

### Factors potentially related to castration-resistance

Three factors were identified in the univariable Cox regression analysis: nadir PSA, total Gleason score, and any concomitant medication (Table 2). Of these, only the nadir PSA and any concomitant medication did not correlate with each other and were subsequently included in the multivariable analysis. In the multivariable analysis, patients with nadir PSA > 2 ng/ml had higher risk of CRPC than those with nadir PSA ≤ 2 ng/ml (HR 21.6, 95% CI 11.7–39.8; *p* < 0.001). Patients taking concomitant medication had higher risk of CRPC than those without concomitant medication (HR 1.8, 95% CI 1.0–3.1; *p* = 0.043).

**Table 2:** Predictive factors of time to CRPC diagnosis in the study population (*N* = 416)

Univariable analysis, variables	Hazard ratio	95% CI	*p* value
Age ≥ 73 years vs < 73 years	1.066	0.651–1.745	0.799
Lowest PSA level reached > 2 ng/ml vs ≤ 2	20.732	11.332–37.929	< 0.001(*)
ECOG performance status 1 vs 0	1.242	0.720–2.143	0.693
ECOG performance status 2–4 vs 0	1.256	0.530–2.974	
D’Amico risk group high vs low-intermediate	1.404	0.694–2.842	0.345
Total Gleason sum ≤ 6 vs ≥ 8	0.439	0.204–0.946	0.014 (*)
Total Gleason sum 3 + 4 vs ≥ 8	0.614	0.312–1.207	
Total Gleason sum 4 + 3 vs ≥ 8	0.340	0.158–0.733	
Concomitant medication (**) yes vs no	1.549	0.888–2.705	0.123
CAB yes vs no	1.218	0.727–2.042	0.454

Multivariable analysis, variables	Hazard ratio	95% CI	*p* value
Concomitant medication (**) yes vs no	1.788	1.018–3.139	0.043 (*)
Lowest PSA level reached > 2 ng/ml vs ≤ 2	21.629	11.745–39.830	< 0.001 (*)

*Values of statistical significance

**Concomitant medications include bicalutamide (*n* = 60), therapeutic radiopharmaceuticals (*n* = 10), abiraterone (*n* = 3), enzalutamide (*n* = 3) and docetaxel (*n* = 1)

## Discussion

In this study, less than 10% of patients non-metastatic at ADT initiation developed CRPC after 3 years of continued treatment. In contrast, CRPC occurred in 40% of patients metastatic at diagnosis during the same time span. Based on previous reports, the percentage of CRPC patients in this study was lower than expected. At the time this study was designed, resistance to LHRHa in less than 3 years had been evaluated only in metastatic patients [19, 20]*.*Our results are aligned with new studies that reported similar rates of CRPC after 3 years of ADT [21, 22]. The risk of CRPC increases in metastatic patients. Our results provide evidence of castration resistance in non-metastatic patients [23].

Differences in definitions of CRPC may mislead physicians to regard ADT as ineffective. Although some loss of effectiveness of ADT is to be expected given a high proportion of androgen-sensitive tumor cells, new combination therapies always include ADT to prevent repopulation of resistant cells [24, 25]. LHRHa have become the most widely used treatment of advanced/metastatic PC on account of their being better accepted than surgical castration, and having less cardiotoxicity than other hormonal treatments with comparable efficacy [17]. Nevertheless, some LHRHa may not reduce serum T below the castration level. A recent study concluded that triptorelin is a more potent agonist, than goserelin and leuprolide, and suggested that triptorelin may be most common in clinical practice in Spain, although the study site selection may not have been representative [26].

For a man with castration-resistant prostate cancer, there is a high probability that this will be the main cause contributing to his death. However, mortality varies in relation to tumor burden assessed as PSA doubling time and PSA at time of CRPC [10]. To date, there are no validated biomarkers of response or toxicity for CRPC other than models and prognostic factors [27, 28]. In our study, the nadir PSA and any concomitant medication were statistically significant predictors of time to CRPC. This is consistent with previous reports that validated concomitant medication as a predictive factor using phase III data [27]. Low molecular weight heparin and warfarin were associated with poorer survival whilst metformin and Cox2 inhibitors were associated with better outcomes [27]. Similarly, ≥ 1% PSA change after ADT was a strong predictor of shorter time to CRPC and overall survival in CRPC metastatic patients [28].

Limitations of this study should include few metastatic patients included (27%), and short follow-up of non-metastatic patients. Also, median time to CRPC was non assessable due to few patients reaching CRPC status. Finally, liquid chromatography with tandem mass spectrometry may not have been used in all local laboratories which may have caused heterogeneity. Another limitation of the study stands in that the influence of previous local treatment (prostatectomy and/or radiotherapy) has not been registered and therefore it could not be evaluated as a potential predictor for development of castration resistance. Finally, the influence of new therapeutic developments in the field of hormone sensitive metastatic PC, such as the use of enzalutamide or apalutamide [29, 30], have not been considered due to the time interval patients were recruited but could likely modify the perspective of CRPC development.

## Conclusion

Despite efforts to develop new therapies, resistance to castration remains a challenge for advanced PC management. This study shows that after 3 years of ADT, nearly 20% of patients developed CRPC; nadir PSA and concomitant medication being predictive factors of the time to CRPC.

## Supplementary Information

Below is the link to the electronic supplementary material.

Supplementary file1 (DOCX 27 kb)

## Data Availability

Where patient data can be anonymized, Ipsen will share all individual participant data that underlie the results reported in this article with qualified researchers who provide a valid research question. Study documents, such as the study protocol and clinical study report, are not always available. Proposals should be submitted to DataSharing@Ipsen.com and will be assessed by a scientific review board. Data are available beginning 6 months and ending 5 years after publication; after this time, only raw data may be available.
