# Supplementary material for: Development of castration resistance in prostate cancer patients treated with luteinizing hormone-releasing hormone analogues (LHRHa): results of the ANARESISTANCE study
Source: World J Urol. 2022 Sep 4;40(10):2459–66. doi: 10.1007/s00345-022-04108-x (PMC9512882; doi:10.1007/s00345-022-04108-x)
Supplement: Supplementary file 1 — Supplementary file1 (DOCX 27 kb) [file 345_2022_4108_MOESM1_ESM.docx]

SUPPLEMENTARY MATERIAL

Supplementary data in this section details additional information as follows:

- Appendix A: Baseline patient distribution based on TNM stage, Gleason score and testosterone level in the enrolled population
- Appendix B: Prostate Specific Antigen levels at each study visit from baseline to month 36, in the Study Population
- Appendix C: Testosterone levels at each study visit from baseline to month 36, in the Study Population
- Appendix D: Study investigators and sites participating in the ANARESISTANCE project

**Appendix A. Percentage of patients in the Per-Protocol Population without CRPC at defined times in the study (according to metastatic status) and with/without CRPC after 3 years of treatment with LHRHa (globally and distributed according to type of androgen blockade).**

|  | | **Per-Protocol Population (n=350)** | |
| --- | --- | --- | --- |
| **No CRPC** |  | **Metastatic (n=90) (*)** | **Non-metastatic (N=239) (*)** |
|  | **6 months** | 100 (95% CI 100-100) | 100 (95% CI 100-100) |
|  | **12 months** | 94.4 (95% CI 87.2-97.6) | 99.2 (95% CI 96.7-99.8) |
|  | **18 months** | 85.6 (95% CI 76.4-91.3) | 97.9 (95% CI 95.0-99.1) |
|  | **24 months** | 77.8 (95% CI 67.7-85.0) | 95.4 (95% CI 91.8-97.4) |
|  | **30 months** | 71.1 (95% CI 60.6-79.3) | 95 (95% CI 91.3-97.1) |
|  | **36 months** | 65.8 (95% CI 54.7-74.8) | 94.5 (95% CI 90.8-96.8) |
| **CRPC vs. No CRPC** |  | **CRPC** | **No CRPC** |
|  | **Globally** | 64 (18.3%) | 286 (81.7%) |
|  | **No-CAB (n=240) (**)** | 42 (17.5%) | 198 (82.5%) |
|  | **CAB (n=102) (**)** | 22 (21.6%) | 80 (78.4%) |

(*) Metastatic status was assessed in 329 patients at initiation of LHRH agonist, in 21 patients the metastatic status baseline was unclassified (n=14) or missing (n=7); (**) Androgen blockade completion was assessed in 342 patients; in 8 patients whether the androgen blockade was complete or not was not specified. CRPC: Castration Resistant Prostate Cancer; CAB: Complete Androgen Blockade.

**Appendix B. Prostate Specific Antigen levels by study visits, baseline to 36-months follow-up, in the Study Population.**

|  | **Screening Visit (V1) (n=400)** | **12 months (V2) (n=370)** | **Change from V2 to V1 (n=358)** | **24 months (V3) (n=324)** | **Change from V3 to V1 (n=316)** | **36 months (V4) (n=292)** | **Change from V4 to V1 (n=283)** |
| --- | --- | --- | --- | --- | --- | --- | --- |
| **Mean (SD)** | 17.9 (36.69) | 2.2 (9.1) | -14.3 (33.24) | 1.1 (5.36) | -14.3 (31.91) | 1.1 (4.32) | -13.0 (28.52) |
| **95% CI Mean** | 14.3-21.5 | 1.2-3.1 | -17.7-(-10.8) | 0.5-1.7 | -17.8 (-10.8) | 0.6-1.6 | -16.3-(-9.6) |
| **Min, Max** | 0, 255 | 0, 76 | -236.7, 57.2 | 0, 76.5 | -237, 68.9 | 0, 50.4 | -236.9, 48.9 |
| **Median** | 6.9 | 0.1 | -6.3 | 0 | -6.5 | 0.1 | -6.5 |
| **Q1, Q3** | 1.3, 16.3 | 0, 0.3 | -14.2, -0.7 | 0, 0.3 | -14.1, -0.7 | 0, 0.5 | -13.4, -0.7 |

(*) Assessed as available in Study Population (N=416); SD: standard deviation; Min: minimum value; Max: Maximum value; Q: quartile.

**Appendix C. Testosterone levels by study visits, baseline to 36-months follow-up, in the Study Population.**

|  | **Screening Visit (V1) (n=284)** | **12 months (V2) (n=323)** | **24 months (V3) (n=280)** | **36 months (V4) (n=263)** |
| --- | --- | --- | --- | --- |
| **Mean (SD)** | 208.6 (228.06) | 37.4 (93.9) | 47.8 (95.47) | 76.6 (129.64) |
| **95% CI Mean** | 182-235.2 | 27.2-47.7 | 36.6-59.1 | 60.9-92.4 |
| **Min, Max** | 0, 966 | 0.0, 908.6 | 0, 656 | 0, 690 |
| **Median** | 120.5 | 15 | 17 | 20.3 |
| **Q1, Q3** | 13, 400 | 4, 30 | 10, 29 | 10, 67 |

(*) Assessed as available in Study Population (N=416); SD: standard deviation; Min: minimum value; Max: Maximum value; Q: quartile.

**Appendix D. ANARESISTANCE study researchers and participating sites**

José Manuel Abascal (Hospital Central Universitario de Asturias, Oviedo); Víctor Barrondo (Hospital Universitario de Basurto, Bilbao); Antonio Benedicto (Hospital Universitario de la Ribera, Alzira); Ana Carballo (Hospital Clínico Universitario de Santiago, Santiago de Compostela); José Ramón Cortiñas (Hospital General de Valladolid); Manuel Fernández (Hospital Universitario del Henares, Coslada); Eduardo Ferrer (Hospital Clínico Universitario de Valencia); Pablo Luís Guzmán (Hospital General Universitario Los Arcos del Mar Menor, Murcia); Miguel Ángel López (Hospital Comarcal Santiago Apóstol, Miranda de Ebro); José Carlos Martínez (Hospital Universitario Nuestra Señora de Candelaria, Santa Cruz de Tenerife); Carlos Olivier (Hospital Universitario de la Princesa, Madrid); Paula Peleteiro (Hospital Clínico Universitario de Santiago, Santiago de Compostela); Pedro Julio Pérez (Hospital Virgen del Puerto, Plasencia); Daniel Pesqueira (Hospital de Povisa, Vigo); José Ponce (Hospital de A Coruña, A Coruña); Manuel Ruibal (Hospital Montecelo, Pontevedra); Josep Segarra (Hospital Universitario Joan XXIII, Tarragona); Eduardo Solsona (Instituto Valenciano de Oncología, Valencia); José Francisco Suárez (Hospital Universitario de Bellvitge, Hospitalet de Llobregat); José Rosa (Hospital Comarcal Santiago Apóstol, Miranda de Ebro); Ángel Tabernero (Hospital Universitario La Paz, Madrid); Félix Vesga (Hospital Universitario de Burgos); Almudena Zapatero (Hospital Universitario de la Princesa, Madrid)
